# Selection of endovascular treatment strategies and analysis of the efficacy of different locations and types of splenic artery aneurysms

**DOI:** 10.1186/s42155-024-00427-9

**Published:** 2024-01-31

**Authors:** Shenjie Wang, Wei Huang, Jingjing Liu, Qin Liu, Ziyin Wang, Qingbing Wang, Qungang Shan, Wenchang Li, Xiaoyi Ding, Zhiyuan Wu, Zhongmin Wang

**Affiliations:** 1 Department of Interventional Radiology, Ruijin Hospital, Shanghai JiaoTong University School of Medicine, 197#, Rui Jin Er Road, Shanghai, 200025 China; 2 College of Health Science and Technology, Shanghai JiaoTong University School of Medicine, Shanghai, China

**Keywords:** Splenic artery, Aneurysm, Endovascular procedures, Stents, Embolization

## Abstract

**Purpose:**

To analyze the selection of endovascular treatment strategies and the efficacy of various locations and types of splenic artery aneurysms (SAAs).

**Methods:**

Sixty-three cases of patients diagnosed with SAA from January 2016 to October 2021 were collected, and their clinical data and follow-up results were analyzed.

**Results:**

Among the 63 patients, 55 had true SAAs, and 8 had false SAAs. The average diameter of the true SAAs was 2.0 ± 0.8 cm. There were 10 cases of intra-aneurysm embolization, 24 cases of intra-aneurysm and aneurysm-bearing artery embolization, 10 cases of bare stent-assisted coil embolization, and 11 cases of stent grafts. The false SAAs had an average diameter of 2.3 ± 1.1 cm. Aneurysm-bearing artery embolization was applied in 5 cases, and stent grafts were applied in 3 cases. The incidence of complications after embolization of the aneurysm-bearing artery was higher (*P* < 0.01). Postembolization syndrome occurred in 10 patients; 7 patients developed splenic infarction to varying degrees, 1 patient had mildly elevated blood amylase, and 1 patient developed splenic necrosis with abscess formation, all of which improved after active treatment. The average length of hospital stay was 5.5 ± 3.2 days. The average follow-up time was 17.2 ± 16.1 months, and the aneurysm cavity of all patients was completely thrombotic.

**Conclusion:**

Endovascular treatments of SAAs are safe and effective. For various locations and types of SAAs, adequate selection of treatment is necessary. Stent grafts are recommended for their safety, economy, practicality, and preservation of the physiological functions of the human body.

## Introduction

Splenic artery aneurysm (SAA) is an uncommon but severely life-threatening vascular disease with a prevalence of approximately 0.8% [1] in the overall population. SAA accounts for 60% of all visceral artery aneurysms and is the third most common intra-abdominal aneurysm, second only to aortic aneurysms and iliac aneurysms [2]. It is defined as an abnormal dilation of the splenic artery over 1 cm in diameter [3]. The significance of splenic artery aneurysm lies in the potential risk for rupture and life threatening hemorrhage which occurs in 10% of cases with a mortality rate of 10–25% in non-pregnant patient and up to 70% during pregnancy [4–7]. The traditional treatment of SAA includes open or laparoscopic surgery to resect the spleen or ligate the blood vessels, but these methods are traumatic and have more complications, a slower postoperative recovery, and higher mortality rates [6, 8–10]. In recent years, endovascular interventional therapy has been developed and has gradually become the initial choice of treatment for SAA due to its advantages of minimal invasiveness, a high success rate, few complications, a fast postoperative recovery, and maximum preservation of splenic blood supply. This study collected the clinical data of 63 SAA patients treated at our hospital, aiming to analyze the corresponding interventional treatment strategies for different types and locations of SAA and their long-term effectiveness, safety, and cost-effectiveness.

## Methods and materials

From January 2016 to October 2021, 63 patients were diagnosed with SAA using computed tomographic angiography (CTA), magnetic resonance angiography (MRA), or digital subtraction angiography (DSA) at our institution, including 55 cases of true SAAs and 8 cases of splenic artery pseudoaneurysms. All patients received endovascular treatment, and their clinical data were collected (Table 1). The retrospective study was approved by the institutional review board, and written informed consent was waived.

**Table 1:** Clinical data of 63 patients with SAAs

Data	True SAAs	False SAAs
**Gender**		
Male	14	6
Female	41	2
**Age (Years)**	57.8 ± 12.0(32–87)	55.1 ± 16.9(32–77)
**Comorbidities**		
Hypertension	25	3
Arteriosclerosis	3	0
Portal hypertension	4	0
**Causes of pseudoaneurysm**		
Pancreatic surgery	/	6
Acute severe pancreatitis	/	1
Trauma	/	1
**Clinical symptoms**		
Yes	14	8
No	41	0
**Location of the aneurysm**		
Initial	4 (involving CT/CHA/SMA)	2 (splenic artery stump)
Proximal	11	5
Mid-section	19	1
Distal	30	0
In the splenic parenchyma	2	0
**Diameter of the aneurysm (cm)**	2.0 ± 0.8 (0.7–4.2)	2.3 ± 1.1 (0.5–3.8)

Among the 55 patients with true SAA, 14 were males and 41 were females, with a mean age of 57.8 ± 12.0 years. A total of 66 true SAAs were found, of which 4 were located at the initial segment of the splenic artery (involving the celiac trunk/CHA/SMA), 11 were at the proximal segment, 19 were at the mid-section, 30 were at the distal segment, and 2 were in the branch artery of the spleen. The mean diameter of the tumor was 2.0 ± 0.8 cm. Fourteen patients had clinical symptoms (abdominal distension, upper left abdominal pain, etc.).

Among the eight patients with splenic artery pseudoaneurysms, one was diagnosed with severe acute pancreatitis (SAP), one was caused by trauma, and the rest underwent pancreatic surgery. The aneurysm was at the splenic artery stump after splenectomy in two cases, at the proximal segment of the splenic artery (SA) in five cases, and at the mid-section in one case.

### Treatment strategy

It is widely agreed that the indications for intervention of SAAs are related to factors that may increase the risk of spontaneous rupture, including [3]: (1) Asymptomatic true SAAs with a diameter ≥ 2 cm, (2) true SAAs with a diameter < 2 cm but gradually increasing during follow-up, (3) true SAAs of any size in women of childbearing age or pregnancy, (4) patients with portal hypertension, (5) true SAAs of any size that should be actively treated when clinical symptoms occur, and (6) splenic artery pseudoaneurysms of any size.

**Fig. 1:**
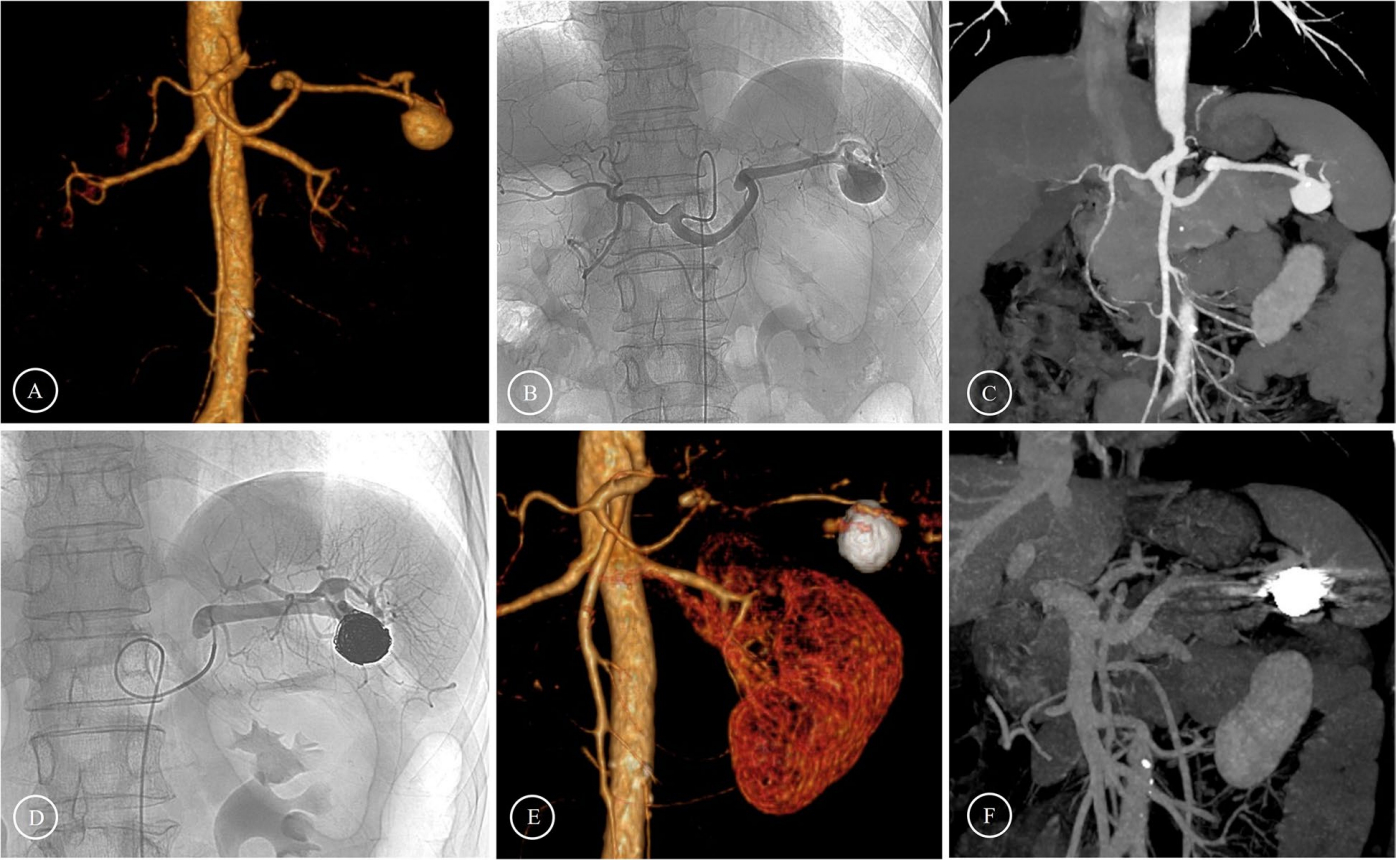
Preoperative and postoperative images of intra-saccular aneurysm coil embolization **A**-**C** Preoperative images showed that the SAA was located at the distal segment of the SA. **D** Coils were implanted in the aneurysm cavity. **E**–**F** The follow-up three months after the operation showed complete formation of thrombosis in the aneurysm cavity

**Fig. 2:**
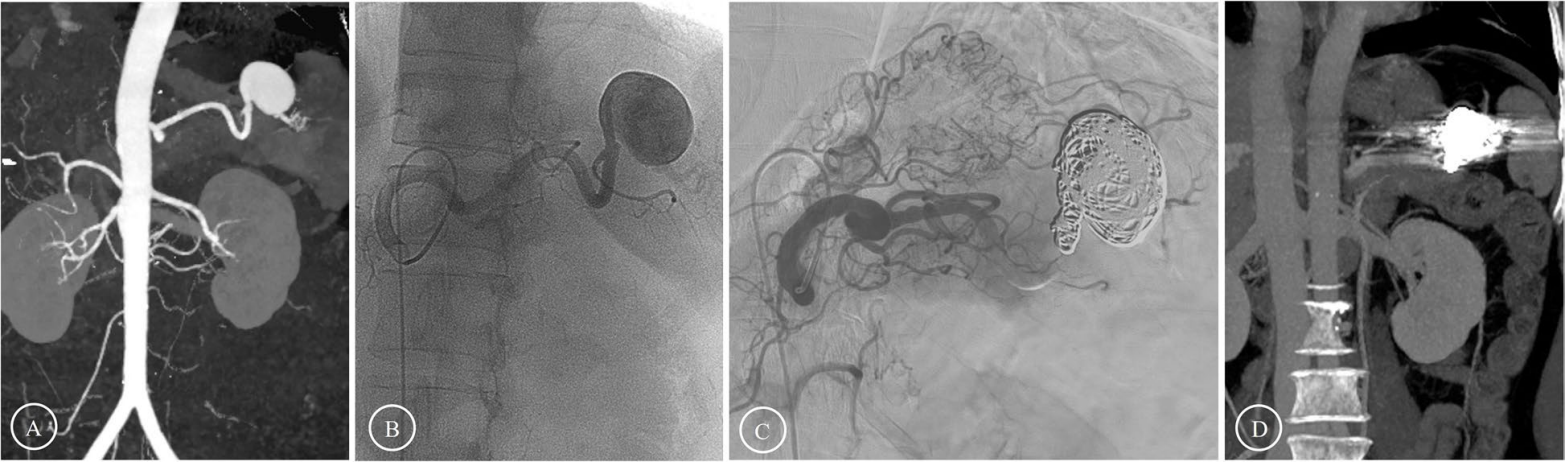
Preoperative and postoperative images of intra-aneurysm and aneurysm-bearing artery coil embolization **A**-**B** Preoperative images showed that a large SAA was located at the distal segment of the SA. **C** Coils were implanted both in the aneurysm cavity and in the aneurysm-bearing arteries, and the collateral circulation was formed immediately to provide blood supply for the spleen. **D** The follow-up three months after the operation showed that no obvious splenic infarction occurred

**Fig. 3:**
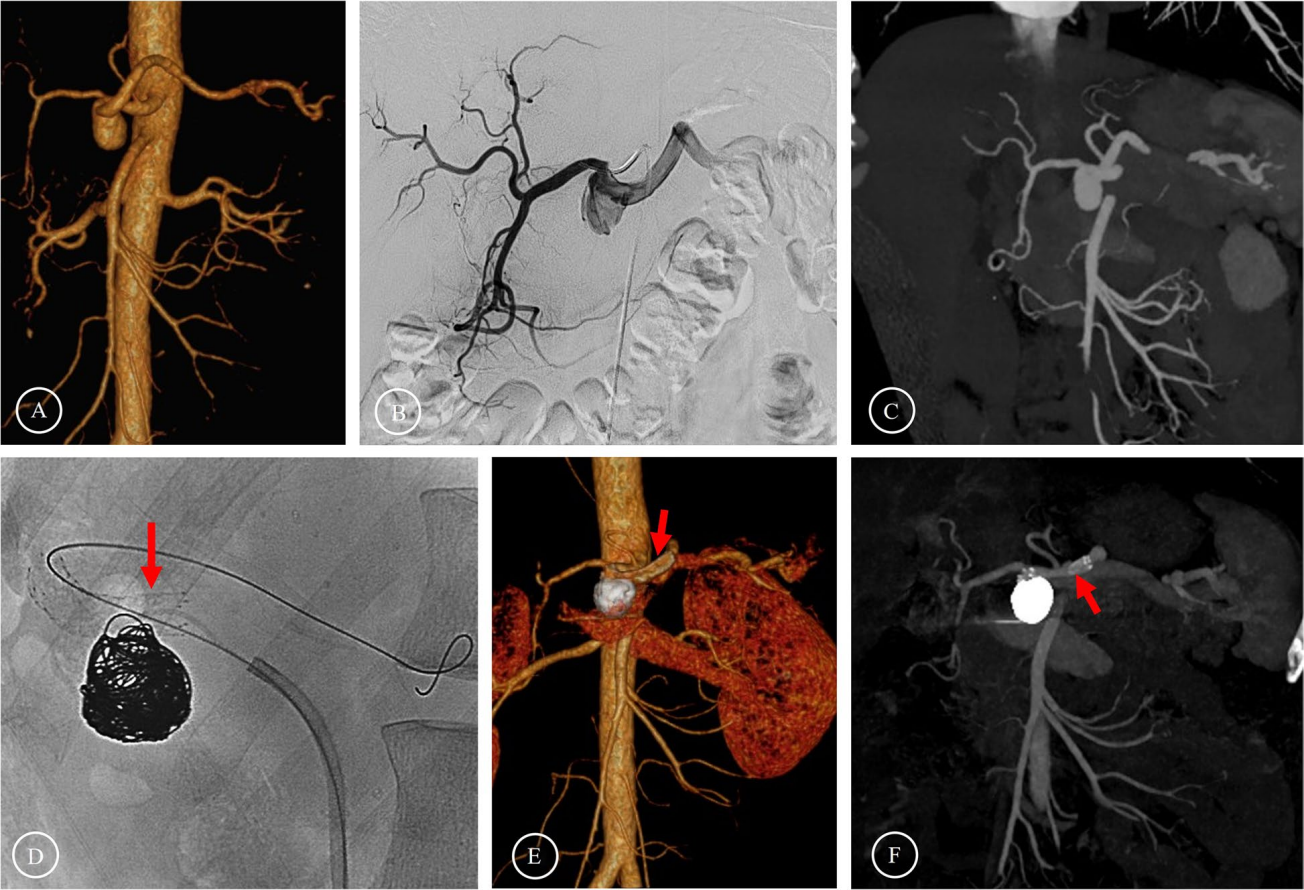
Preoperative and postoperative images of bare stent-assisted coil embolization **A**-**C** Preoperative images showed that the SAA was located at the initial segment of the SA and involved the celiac trunk. **D** Coils were implanted in the aneurysm cavity after the bare stent was released. **E**–**F** The follow-up one year after the operation showed that the formation of thrombosis in the aneurysm cavity was complete and the blood flow in the bare stent was unobstructed. (The red arrows indicate the bare stent.)

**Fig. 4:**
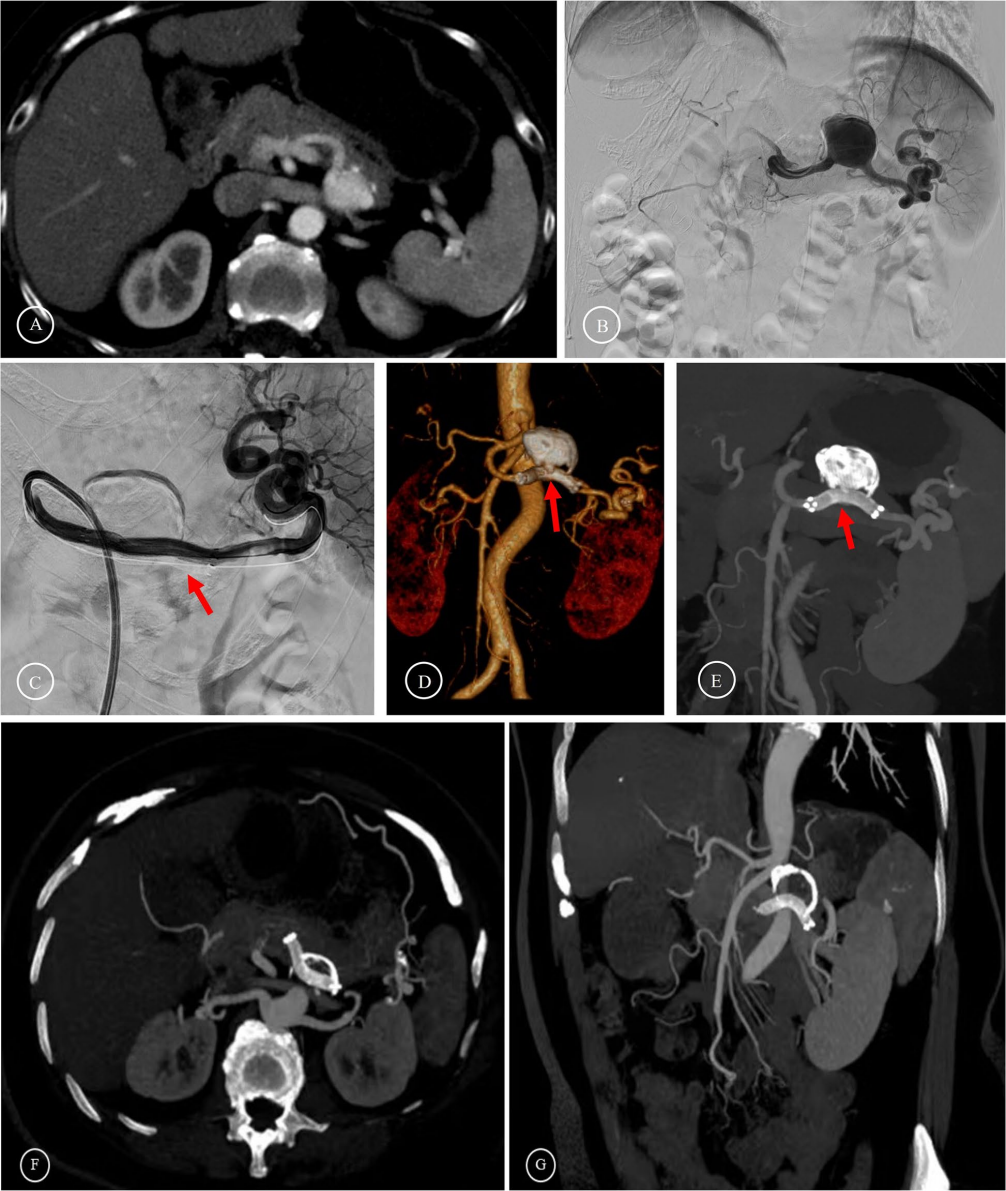
Preoperative and postoperative images of stent graft endovascular exclusion **A**-**B** Preoperative images showed that the SAA was located at the mid-section of the SA. **C** The stent graft (Viabahn) was released to isolate the aneurysm. **D**-**E** The follow-up two months after the operation showed that the blood flow in the stent graft was unobstructed. (The red arrows indicate the stent graft.) **F**-**G** The follow-up three years after the operation showed the calcium wrap around the aneurysm

The patient was placed in the supine position, the right inguinal puncture point was locally anesthetized with 2% lidocaine, the right femoral artery was punctured using the Seldinger technique, and the catheter sheath (Terumo, Tokyo, Japan) was inserted. Under the guidance of DSA, the 4F RH catheter (Cordis, Johnson & Johnson, New Jersey) was intubated to the celiac trunk and superior mesenteric artery (SMA) for imaging to clarify the location, shape, extent of involvement, tumor-bearing arteries, collateral vessels, and spleen imaging. According to the imaging results, endovascular treatment methods are divided into the following five types: (1) Intra-saccular aneurysm coil embolization: For true SAAs with narrow necks, the treatment effect can be achieved by directly filling the aneurysm cavity with coils (Interlock 0.018-in, Boston Scientific, Marlborough, Massachusetts). During the operation, care should be taken to select a coil with a suitable diameter to avoid the aneurysm from rupturing while embolizing tightly. At the same time, the release should be accurate to prevent the coil from shifting (Fig. 1). (2) Aneurysm-bearing artery coil embolization: For true SAAs with a large diameter, the inflow and outflow tracts of the aneurysm can be embolized with coils regardless of whether the aneurysm cavity is filled or not. It can be considered satisfactory when no contrast agent is filled in the aneurysm and the outflow tract does not develop during postprocedural angiography. (3) Intra-aneurysm and aneurysm-bearing artery coil embolization: In actual clinical practice, most cases are combined applications of the above two methods (Fig. 2). (4) Bare stent-assisted coil embolization: It is suitable for aneurysms that are located close to the beginning of the splenic artery and involve the celiac trunk, CHA or SMA, as well as for aneurysms with a wide neck. Bare stent-assisted coil embolization can preserve important blood vessels and ensure that the coils do not shift. After confirming the location and size of the aneurysm through CTA and DSA, a bare stent of appropriate size is selected (Express SD, 0.014-in/0.018-in, Boston Scientific, Marlborough, Massachusetts and Pulsar-18, Biotronik, Berlin, Germany). The coils are released by passing the microcatheter through the mesh of the bare stent or through the microcatheter reserved in the aneurysm before the stent is released (Fig. 3). (5) Stent graft endovascular exclusion: It is suitable for true aneurysms that are in the main trunk of the splenic artery with sufficient anchoring length from the beginning of the splenic artery and have relatively straight blood vessels. It is also suitable for the treatment of pseudoaneurysms. After confirming the position of the aneurysm through angiography, a stent graft (Viabahn: W.L. Gore & Associates, Flagstaff, Arizona) with an appropriate diameter (10% larger than the diameter of the aneurysm-bearing artery) and length (at least 1 cm beyond both ends of the aneurysm neck) is selected. It can be considered satisfactory when no contrast agent is filled in the aneurysm and the blood flow of the splenic artery is smooth during postprocedural angiography (Fig. 4).

### Postoperative treatment and follow-up

Close observation of postembolization syndromes, such as abdominal pain, nausea, vomiting and fever, and routine blood tests and amylase tests were performed on the first day after the operation. For patients receiving coil embolization, antibiotics such as cephalosporins are routinely used to prevent the formation of splenic abscesses. For patients with true SAA who received stent implantation, antiplatelet therapy (aspirin 100 mg/d and clopidogrel 75 mg/d) was given for at least three months after the operation. Patients with pseudoaneurysms who received stent implantation were treated the same as true SAA after ensuring that no other high-risk bleeding factors existed.

CTA was reexamined one month after the operation, and follow-up visits were performed in outpatient clinics or by telephone.

## Results

### Location of the SAAs and selection of treatment

Among the 55 patients with true SAAs, a total of 66 aneurysms were found, of which four were located at the initial segment of the splenic artery (celiac trunk/CHA/SMA involved), and all underwent bare stent-assisted coil embolization. Eleven aneurysms were at the proximal segment, and no significant difference in the choice of treatment methods was found. Nineteen aneurysms were located at the mid-section of the splenic artery, and nearly half of them were chosen for application of the stent grafts. Thirty aneurysms were at the distal segment, except for two cases in which bare stent-assisted coil embolization was performed, and the rest were treated with intra-saccular aneurysm coil embolization. Two were in the branch arteries of the spleen, and both were treated with intra-aneurysm and aneurysm-bearing artery coil embolization (Table 2).

**Table 2:** Locations of 66 true SAAs and the selection of treatment

	Intra-saccular aneurysm coil embolization	Intra-aneurysm and aneurysm-bearing artery coil embolization	Bare stent- assisted coil embolization	Stent graft endovascular exclusion
Initial (involving CT/CHA/SMA)	0	0	***4(100%)***	0
Proximal	2(18.18%)	4(36.37%)	3(27.27%)	2(18.18%)
Mid-section	3(15.79%)	6(31.58%)	1(5.26%)	***9(47.37%)***
Distal	7(23.33%)	***21(70%)***	2(6.67%)	0
In the splenic parenchyma	0	***2(100%)***	0	0

Of the eight splenic artery pseudoaneurysms, two were located at the splenic artery stump and underwent aneurysm-bearing artery coil embolization and stent graft endovascular exclusion. Five were located at the proximal segment, of which three received aneurysm-bearing artery coil embolization and two underwent stent graft endovascular exclusion. Only one was in the mid-section of the SA and underwent aneurysm-bearing artery coil embolization.

### Surgical results and postoperative complications

All 63 cases of endovascular treatment were successfully completed, and the operation success rate was 100%.

Among the 55 cases with true SAA, 10 cases were treated with intra-saccular aneurysm coil embolization, 24 cases with intra-aneurysm and aneurysm-bearing artery coil embolization, 10 cases with bare stent-assisted coil embolization, and 11 cases with stent graft endovascular exclusion. Nine patients developed postembolization syndrome, in which abdominal pain and low fever were all relieved after symptomatic treatment with NSAIDs. Seven patients treated with intra-aneurysm and aneurysm-bearing artery coil embolization developed splenic infarction to varying degrees, without splenic abscess or abdominal infection. Another patient treated with bare stent-assisted coil embolization showed a slight increase in blood amylase on the first day after the operation and returned to normal on the third day. Eleven patients treated with stent graft endovascular exclusion did not experience any postoperative complications (Table 3). The difference between these four treatment groups was statistically significant (*P* < 0.01).

**Table 3:** Outcomes and post procedure complications of 55 patients with true SAAs

	Intra-saccular aneurysm coil embolization	Intra-aneurysm and aneurysm-bearing artery coil embolization	Bare stent- assisted coil embolization	Stent graft endovascular exclusion	Total	Chi-Square	*P Value*
Cases	10	24	10	11	55	/	/
Postembolization syndrome	1	6	2	0	9		
Splenic infarction	0	7	0	0	7		
Increased blood amylase	0	0	1	0	1		
Total complications	1 (10.0%)	13 (54.2%)	3 (30.0%)	0	17 (30.9%)	13.012	0.002
Length of hospital stay (d)	4.4 ± 2.1	5.6 ± 3.7	6.2 ± 3.6	5.6 ± 2.7	5.5 ± 3.2	1.339	0.720

Obviously, the postoperative complication rate of aneurysm-bearing artery embolization was significantly higher than that of the other three treatment methods. Therefore, the aneurysm-bearing artery non-embolized group compared with the aneurysm-bearing artery embolized group showed a higher incidence of complications after embolization of the aneurysm-bearing artery, and the difference was statistically significant (*P* = 0.003) (Table 4). The aneurysm-bearing artery non-embolized group was divided into the stent graft group and the coil group, and the subgroup analysis showed that the difference between the two groups was not statistically significant (*P* = 0.303) (Table 5).

**Table 4:** Complications in the embolized and non-embolized groups

	Aneurysm-bearing artery embolized	Aneurysm-bearing artery non-embolized ^a^	Chi-Square	*P value*
Cases	24	31	/	*/*
Postembolization syndrome	6	3		
Splenic infarction	7	0		
Increased blood amylase	0	1		
Total complications	13	4	8.940	0.003

^a^The aneurysm-bearing artery non-embolized group included intra-saccular aneurysm coil embolization, bare stent-assisted coil embolization and stent graft endovascular exclusion

**Table 5:** Complications in the stent graft group and coil group

	Stent graft	Coil ^b^	Chi-Square	Residual	*P value*
Cases	11	20	/	*/*	*/*
Postembolization syndrome	0	3			
Splenic infarction	0	0			
Increased blood amylase	0	1			
Total complications	0	4	1.060	1.6	0.303

^b^The coil group included intra-saccular aneurysm coil embolization and bare stent-assisted coil embolization

Among the eight patients with splenic artery pseudoaneurysms, five were treated with aneurysm-bearing artery coil embolization, and three were treated with stent graft endovascular exclusion. One patient developed postembolization syndrome, which improved after symptomatic treatment with NSAIDS. Another patient who previously underwent a pancreaticoduodenectomy and subtotal gastrectomy had the gastroduodenal artery (GDA), left gastric artery (LGA), and gastroepiploic arch ligated during surgery, and effective collateral circulation could not be established after splenic artery embolization, leading to splenic necrosis with abscess formation. After CT-guided percutaneous drainage and systemic antibiotic treatment, the intra-abdominal infection was effectively controlled.

The average length of hospital stay (LHS) of the 55 patients with true SAA was 5.5 ± 3.2 days, and there was no significant difference between the different treatment groups (*P* = 0.720). The patients with splenic artery pseudoaneurysms generally had critical diseases, most of which required ICU monitoring or even multiple surgical treatments. This characteristic contributed too many interfering factors, so the LHS of these patients was not included in this study.

### Follow-up

A CTA reexamination was performed one month after the operation. There was no filling of contrast agent in the aneurysm cavities of all patients. The postoperative follow-up period was 1–68 months, with an average of 17.2 ± 16.1 months. Thrombosis in the aneurysms was obvious in the patients undergoing coil embolization, the blood flow in the stent was unobstructed in the patients undergoing stent implantation, and no thrombosis or occlusion was observed in the stents. Except for one patient who was lost to follow-up four months after the operation, one patient died of cholangiocarcinoma after ten months of follow-up, and the aneurysm of another patient treated by intra-saccular aneurysm coil embolization ruptured 6 months after the operation and accepted emergency open surgery. No aneurysm enlargement, rupture, or recurrence was observed during the follow-up period of the remaining 60 patients, and no interventional treatment was performed again.

## Discussion

In 1770, Beaussier first reported SAA in cadavers [11]. It accounts for approximately 60% of all visceral aneurysms and is four times more common in females than in males [12–14]. In addition to congenital fibromuscular dysplasia and arterial wall injury, the pathogenesis of SAAs is usually related to increased splenic arterial blood flow, such as multiple pregnancy, arteriovenous fistulas and malformations, and portal hypertension [15]. Previous studies have shown that increased blood flow in the splenic artery can lead to irreversible damage to the arterial media, which leads to atrophy and calcification of the wall muscle [16]. Other studies have summarized the risk factors for SAAs, including hypertension, atherosclerosis, liver cirrhosis, and diabetes [17–20]. It was widely agreed that the risk of rupture of SAAs with a diameter of ≥ 2 cm was significantly increased [21–23]. Once the aneurysm ruptures, the fatality rate can reach as high as 40% [24].

The selection of endovascular treatments for SAAs varies with the location and type of aneurysm. The results of this study fully reflect that choosing the corresponding treatment methods can result in ideal treatment effects.

### Stent grafts are highly recommended for true SAAs located at the proximal or mid-section of the splenic artery

According to the results of this study, the incidence of complications such as splenic infarction after embolization of the aneurysm-bearing artery is significantly increased. Therefore, we recommend that the aneurysm-bearing artery be preserved to the greatest extent, and treatments that do not affect blood flow in the splenic artery should be selected. Among the three treatments for preserving the aneurysm-bearing artery, we strongly recommend choosing the stent graft as the first choice, especially in cases where the aneurysm is located at the proximal but not in the initial segment or mid-section of the splenic artery and has good vascular pass ability. Although it may be due to the small amount of overall data, the data in this study did not show a significant difference between the stent graft and the coil treatment, but the superiority of the stent graft was not seen in the coil. The stent graft still allows blood flow through the stent while isolating the aneurysm, thereby ensuring the patency of the splenic artery, and preserving splenic perfusion, thus greatly reducing the risk of splenic infarction, and supporting the normal physiological functions of the human body. Additionally, the application of stent graft can reduce the use of coils to a considerable extent, reduce medical costs, and decrease operation time. In addition, the 0.018-in stent graft has better pass ability due to the smaller outer diameter of the delivery system and is most suitable for SAAs at the proximal segment and mid-section. Moreover, in this study, a patient who received intra-saccular aneurysm coil embolization had a sudden aneurysm rupture six months after the operation and underwent emergency surgery. Therefore, an exceedingly small probability of rupture may still exist after embolization of the aneurysm cavity with coils. The application of stent grafts does not appear to have similar consequences.

### Bare stent-assisted coil embolization is more suitable for true SAAs at the initial segment of the splenic artery

For true SAAs with wide necks or involving important blood vessels at the initial segment of the splenic artery, we recommend routine treatment with bare stent-assisted coil embolization. The reason why a stent graft is not recommended is that the anchoring part of the proximal end of the covered stent may block important arteries such as the celiac trunk/CHA/SMA. Forcible preservation of these arteries may result in insufficient anchoring distance of the proximal end of the stent and cause endoleakage. Intra-saccular aneurysm coil embolization is also inappropriate, as the coils will face the impact of high-speed blood flow of the inflow tract, and they may not stay stable in the aneurysm. Once the coils fall off to the distal end of the artery, they can cause ectopic embolism. The advantage of bare stents is that, while covering the aneurysm and the inflow and outflow tracts, the blood flow can pass through the meshes on the stent, thus ensuring that important blood vessels are not mistakenly covered. After the bare stent is implanted, coils can be introduced with a microcatheter placed in the aneurysm cavity in advance. Meanwhile, the bare stent acts as a barrier, blocking the embolism material completely in the aneurysm cavity without shifting [25].

### Coils are the only choice for true SAAs in the distal segment of the splenic artery and the splenic parenchyma

Because the splenic artery is often long and twisted, for true SAAs in the distal segment of the splenic artery and the splenic parenchyma, the pass ability of coils makes it almost the only option for treatment. Studies have reported that the success rate of coil embolization is 90%-100% [19, 26, 27]. One study included 90 cases of visceral artery aneurysms (VAAs) at various locations. The success rate of coil embolization was 98%, and the perioperative (30 d) mortality rate was only 8.3% [19]. To ensure the safety and effectiveness of embolization, we recommend using detachable coils with fibers. Compared with ordinary coils, synthetic fibers are designed for greater thrombogenicity. The detachable system allows the coil to be removed before finally entering the blood vessel, thus improving the safety of the operation.

### Treatment for pseudoaneurysms should be fast and precise

For splenic artery pseudoaneurysms, the focus of the treatment strategy is different from that for true SAAs. As these patients often suffer from trauma, pancreatic surgery, acute pancreatitis, and other incentives, they are in critical condition and may undergo hemorrhagic shock at any moment. Therefore, rapidly blocking the blood flow and stopping the bleeding are the top priorities of pseudoaneurysm treatment. For this reason, surgeons need to carefully evaluate the application of coils or stent grafts based on intraoperative angiography. The main goal is to save lives. If necessary, even if we use coils or stent grafts to completely block the splenic artery, the communicating branches of the short gastric artery and dorsal pancreatic artery can quickly provide blood supply for the spleen through collateral circulation, so severe splenic infarction is generally rare. We believe that the key points of pseudoaneurysm treatment are to be “fast” and’precise’, and we should not hesitate because of concerns about possible postoperative complications. Only by 'cutting the Gordian knot' can the patient be rescued from death.

## Conclusion

Endovascular treatments for SAA are safe and effective, and for various locations and types of aneurysms, adequate evaluation and selection of appropriate interventional treatment methods can achieve ideal therapeutic effects and better long-term prognoses. If conditions permit, stent grafts are the most ideal choice due to their safety, practicality, cost-effectiveness, and support of the physiological functions of the human body.

## Data Availability

All data generated or analyzed during this study are included in this published article.

## Abbreviations

SAA: Splenic artery aneurysm
CTA: Computed tomographic angiography
MRA: Magnetic resonance angiography
DSA: Digital subtraction angiography
CHA: Common hepatic artery
SMA: Superior mesenteric artery
SA: Splenic artery
SAP: Severe acute pancreatitis
NSAIDs: Nonsteroidal anti-inflammatory drugs
GDA: Gastroduodenal artery
LGA: Left gastric artery
LHS: Length of hospital stay
SVS: The Society for Vascular Surgery
VAA: Visceral artery aneurysms
